# Riboflavin: a potentially important skin pigment in reptiles

**DOI:** 10.1098/rsos.261008

**Published:** 2026-09-30

**Authors:** Athanasia C. Tzika, Adrian Lutz, Maria Thaker, Laure Menin, Devi Stuart-Fox

**Affiliations:** 1 University of Geneva, Geneva, Switzerland; 2 School of BioSciences, The University of Melbourne, Melbourne, Victoria, Australia; 3 Centre for Ecological Sciences, Indian Institute of Science, Bangalore, Karnataka, India; 4 École polytechnique fédérale de Lausanne, Lausanne, Switzerland

**Keywords:** skin pigment, chromatophore, carotenoid, flavin, pteridine, xanthophore, biochemistry, cellular biology, physiology

## Abstract

Pigments not only contribute to the extraordinary diversity of animal coloration but also play vital physiological roles. Riboflavin, or vitamin B2, is a macronutrient essential for cellular metabolism and growth, and is a well-known yellow pigment produced industrially for a wide range of applications, both as a colourant and dietary supplement. Remarkably, however, its role in animal skin coloration has been largely overlooked. Using quantitative mass spectrometry, we show that high riboflavin concentrations correspond to yellow hues in the skin of two species of lizard and one snake species. The comparison of riboflavin concentrations in different skin colours within individuals provides compelling evidence that riboflavin can contribute to skin pigmentation in reptiles beyond its general role in cellular metabolism. The role of riboflavin was further corroborated by high concentrations of riboflavin in yellow blotches of the Snow morph of the corn snake. In this mutant, xanthophore pigment cells, which in wild-type corn snakes contain pteridine pigments, are heavily impacted, and the overall amount of yellow pteridines is low. Like carotenoids, riboflavin must be acquired through diet and has powerful antioxidant properties. Thus, its use in coloration may have unrecognized ecological and evolutionary implications.

## 1 Introduction

In ectothermic vertebrates (fishes, amphibians and reptiles), red to yellow skin colours are traditionally thought to be produced by two biochemically distinct classes of pigment: carotenoids, which are acquired through the diet, and pteridines, which are synthesized de novo [1–3]. Carotenoids have received a great deal of research attention because carotenoid acquisition and metabolism have important eco-evolutionary implications [4–6]. For example, dietary availability may be limiting, and carotenoid metabolism is intrinsically linked to vital cellular processes, which may explain the honesty of colour signals [4–6]. However, in reptiles, the contribution of carotenoids appears to vary substantially among species [7–11]. For example, among 27 species of agamid lizards that we examined previously, carotenoid concentrations were not associated with skin colour, whereas the concentration of pteridines was associated with skin hue, saturation and luminance [3]. Furthermore, in most snakes examined to date, carotenoids may contribute little to observed skin colour [12, 13].

Recent reviews of colour mechanisms in reptiles or snakes primarily discuss these well-studied pigment classes [1,12]. However, limited evidence suggests that riboflavin (vitamin B2), may also contribute to reptile coloration. Although riboflavin belongs to the group of flavin compounds, it is sometimes grouped broadly with pteridines and their derivatives owing to its fused ring scaffold. However, riboflavin and pteridines differ substantially in their biochemical properties and biological functions. For example, pteridines are synthesized de novo and are soluble in acidic or alkaline solutions, whereas riboflavin is a water-soluble micronutrient produced by microorganisms (bacteria and yeast), with no de novo synthesis pathway in vertebrates [14]. In this regard, riboflavin resembles carotenoids, which must also be acquired from the diet and are antioxidants with essential physiological roles [5]. Thus, if riboflavin contributes to skin coloration, it could have eco-evolutionary implications analogous to those driving decades of research on carotenoid-based signals.

Studies in the 1950s and 1960s [15,16] reported high concentrations of the yellow riboflavin in the green skin of the African spotted bush snake (*Philothamnus semivariegatus*) and the boomslang (*Dispholidus typus*) [15], as well as the yellow bands of the banded krait (*Bungarus fasciatus*) [16]. Riboflavin was also detected in several other snake species with more conspicuous skin coloration, including several colubrids [17] and the Indian python (*Python molurus*) [16], and in the skin of the Japanese gecko (*Gekko japonicus*) [17]. Interestingly, studies focusing on frog [17,18] and fish [19] skin reported the presence of riboflavin either in isolated melanosomes (which typically contain black melanin pigment) or associated with skin collagen, where its functional significance remained unclear. Subsequent research revealed the crucial role of riboflavin in cellular metabolism and growth [14], supporting the view that its presence in skin reflected its general metabolic role rather than a specific contribution to pigmentation. Consequently, riboflavin was largely overlooked as a skin colourant in ectothermic vertebrates, despite these early reports of differences in concentration between species. Moreover, the few studies that have considered riboflavin as a skin pigment have examined it qualitatively rather than quantitatively (e.g. several freshwater turtles [20] and a teleost fish [21]); thus, its contribution relative to yellow carotenoid and pteridine pigments remains uncertain.

**Figure 1:**
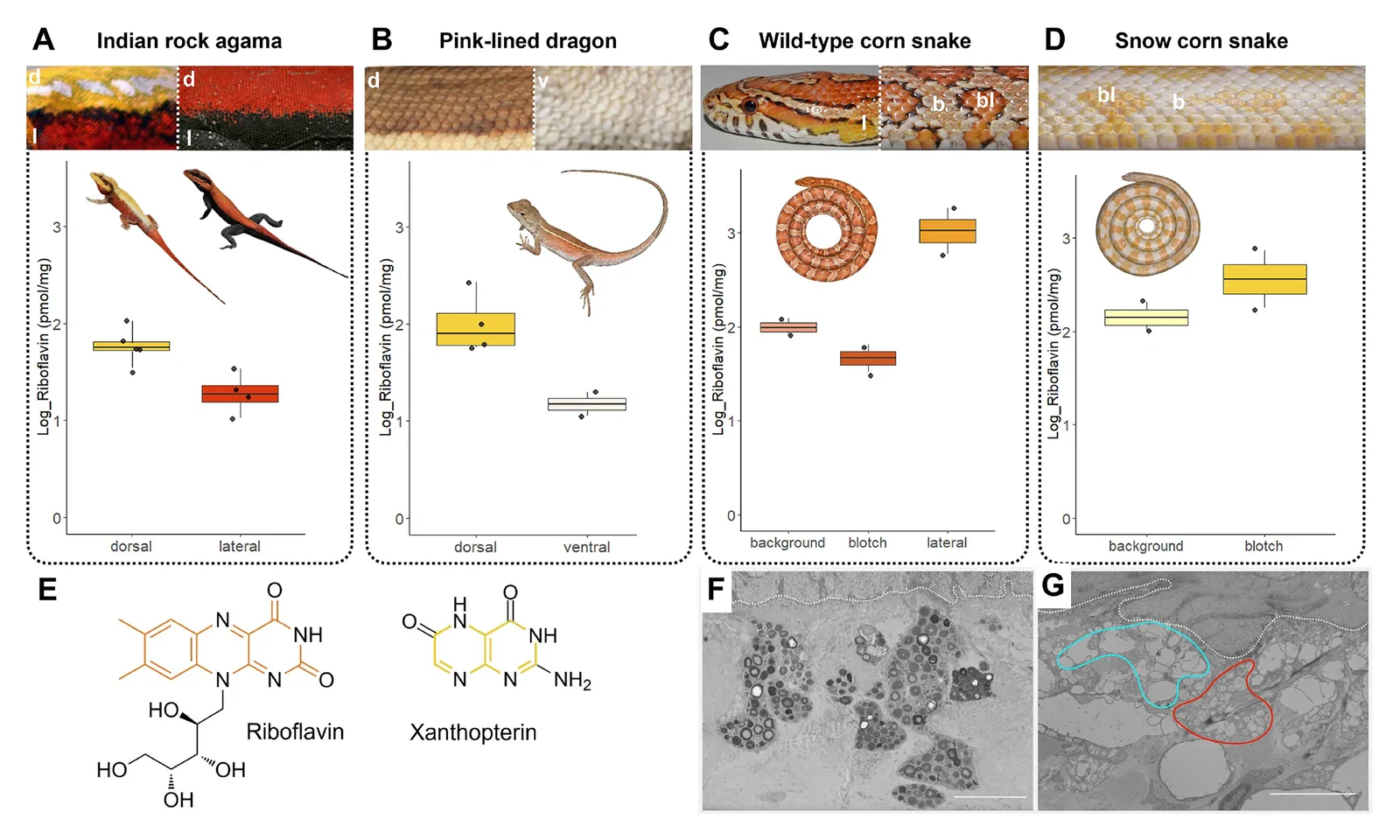
High riboflavin concentration is associated with yellow skin. Riboflavin concentrations are shown as box and whisker plots in which the horizontal line indicates the median, the box indicates the interquartile range, and whiskers indicate minimum and maximum values excluding outliers. (A) Dorsal and lateral skin of the Indian rock agama (photos on top illustrate the skin colour change during male–female interactions). (B) Different body regions in the pink-lined dragon. (C) Wild-type and (D) Snow colour morphs of the corn snake. d: dorsal, l: lateral, v: ventral, b: background and bl: blotch. Box colours correspond approximately to the associated skin colours. (E) Chemical structure of riboflavin and xanthopterin (a yellow pteridine), showing the tricyclic isoalloxazine ring that characterizes flavins in orange and the dicyclic fused pyrazine and pyrimidine ring that characterizes pteridines in yellow. (F) Electron micrographs of xanthosomes in wild-type skin with concentric lamellae, typical of pteridine-containing vesicles (i.e. pterinosomes). (G) In Snow skin, we observe abnormal xanthosomes with amorphous material (examples outlined in red solid line) and enlarged vesicles (examples outlined in cyan solid line). The dashed white lines mark the dermis–epidermis boundary; scale bars, 5 μm.

Here, we examine the contribution of riboflavin to yellow and orange skin colour in two agamid lizard species and a snake species (figure 1). Using high-performance liquid chromatography–mass spectrometry (LC-MS), we precisely quantified concentrations of carotenoids, pteridines and riboflavin in the skin of two species of lizards from the family Agamidae, the Indian rock agama (*Psammophilus dorsalis*) and the pink-lined dragon (*Diporiphora linga*) and two colour morphs of the corn snake (*Pantherophis guttatus*; figure 1A–D). The three species span a broad evolutionary range, with Colubridae snakes and Agamidae lizards diverging approximately 161 million years ago [22]. By comparing pigment concentrations between colour patches within individuals, we reveal how riboflavin contributes to skin coloration beyond its general role in cell metabolism. Additionally, we exploit the Snow morph of the corn snake, which displays yellow blotches without the orange–red hues typical of wild-type, to isolate the contribution of riboflavin from other yellow pigments. Together, our data confirm that riboflavin is a potentially important yellow skin pigment in reptiles and highlight the need to consider its contribution in future studies of vertebrate pigmentation.

## 2 Methods

### 2.1 Study species and sampling

This study was designed as a discovery-level biochemical analysis; therefore, to minimize animal use, we sampled the smallest number of individuals required to identify pigments and compare colour patches within individuals. For each species or morph, we sampled distinctly coloured skin patches rather than specific locations owing to the differences among them in colour patterning and coloration intensity.

We sampled skin from wild-caught pink-lined dragons (two males) from western South Australia in December 2022, and the Indian rock agama (two males) from the vicinity of Bangalore, India, in April–June 2024. Corn snakes were housed and bred at the LANE animal facility running under the veterinary cantonal permit no. 1008. Male and female corn snakes have similar colours. We sampled a female wildtype and a male Snow. Samples from each body region were stored at –80°C or preserved in methanol for subsequent extractions.

Males of the Indian rock agama rapidly change colour in the presence of rivals and prospective mates [23]. The upper half of the body (dorsal stripe and upper lip) is yellow in the presence of rivals and red when courting females, whereas the lower half of the body (thick lateral stripe and throat) is red in the presence of rivals and black when courting females. Accordingly, we compared concentrations of riboflavin in the dorsal stripe and upper lip, which contain yellow, versus the lateral stripe and throat, which do not contain yellow (figure 1A). Pigment concentrations were quantified for each of the four body regions for both individuals. The yellow regions remain in the yellow state after death (i.e. when sampled), although the colour change would not affect results because at this time scale it involves changes in dispersion/aggregation, rather than absolute concentration, of pigments in skin.

The pink-lined dragon has a thick yellow–brown dorsal stripe and a thin pale yellow dorsolateral stripe, whereas the throat is creamy-white (figure 1B). Therefore, we compared concentrations of riboflavin in the two dorsal stripes (dorsal) with those of the throat (ventral).

Wild-type corn snakes display red blotches on an orange background (figure 1C), whereas the Snow morph, a double amelanistic [24] and anerythristic mutant, has yellow blotches on a cream background (figure 1D). For the corn snake morphs, we took replicate skin samples (samples of each colour patch from different parts of the body) for an individual of each morph. For the wild-type, we took samples of the brick-red dorsal blotches, orange background and yellow lateral blotches near the head. For the Snow morph, we took samples of the yellow blotches and cream background.

**Figure 2:**
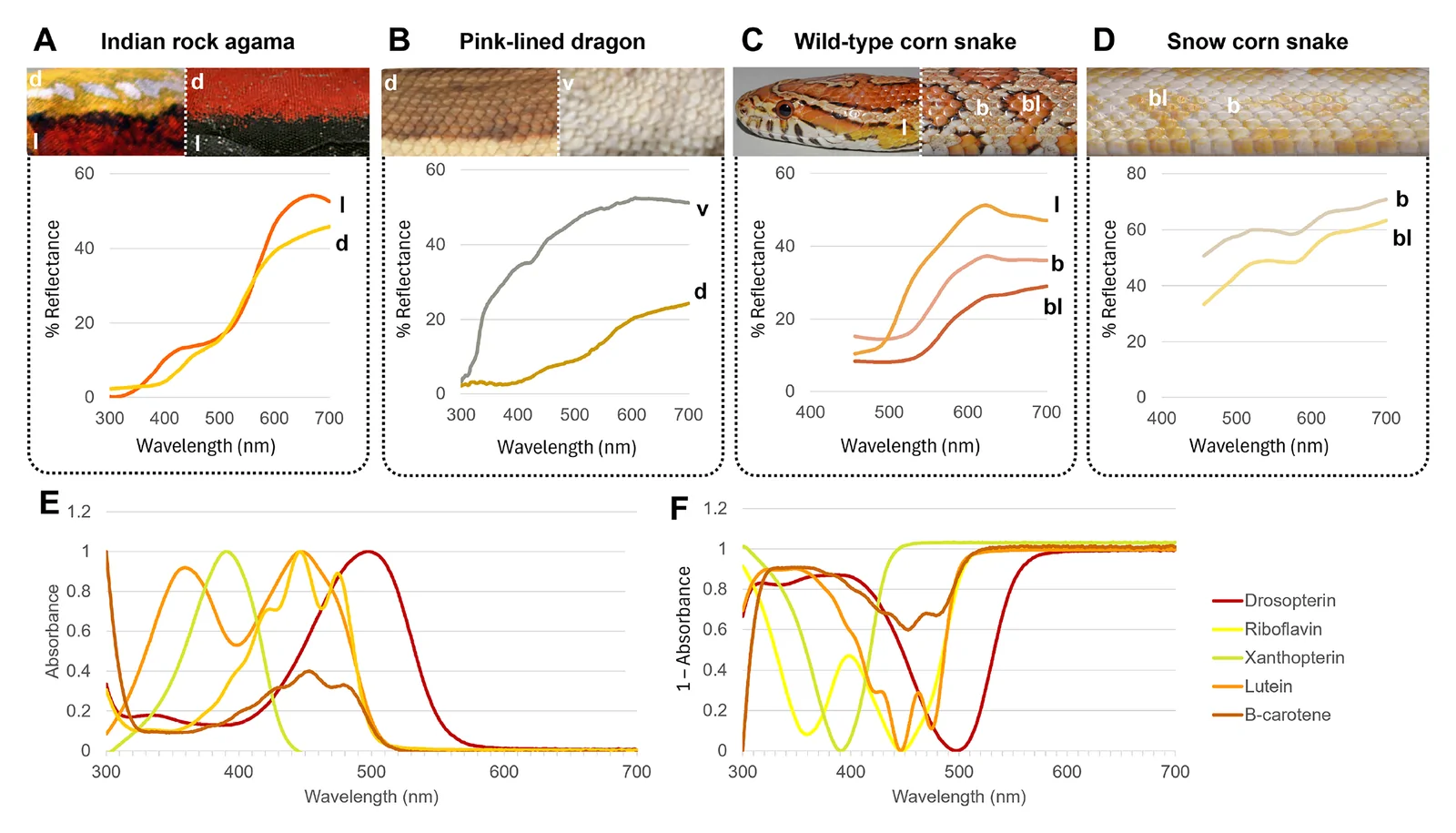
Reflectance profiles of the colour patches for which we quantified pigments. (A) Dorsal (yellow–orange state) and lateral (red state) of the Indian rock agama (photos on top illustrate the skin colour change during male–male interactions [23]). (B) Different body regions in the pink-lined dragon. (C) Wild-type and (D) Snow colour morphs of the corn snake. d: dorsal, l: lateral, v: ventral, b: background and bl: blotch. (E) Absorbance profiles of the yellow to red pigments found in the selected species. Panel (F) shows the same data as in (E), but the *y*-axis is shown as 1 minus absorbance to facilitate comparison with the skin reflectance spectra (this is equivalent to assuming pigments are underlain by a perfect reflector). Note the similar cut-off value of wavelength at close to 500 nm for lutein (carotenoid) and riboflavin.

For all samples, we took spectrophotometry measurements of the skin samples *in vivo* (figure 2A–D). For the pink-lined dragon, we took measurements with a JAZ EL–200 spectrometer with an inbuilt JAZ–PX pulsed xenon light source (Ocean Optics, Dunedin, FL). For the Indian rock agama, measurements were taken with a JAZA2474 and PX light source (Ocean Optics) as detailed in [23]. For both species, the measurements were taken at a 45° angle relative to the surface and were calibrated using a diffuse 99% spectralon reflectance standard (Labsphere). For the corn snakes, measurements were taken with a hyperspectral camera (Cubert GmbH, FireflEYE S185 SE).

### 2.2 Reptile skin pigment quantitation

We further adapted LC-MS and extraction methodologies used in [3] to include riboflavin (Pubchem CID: 805117) and quantitate drosopterin (Pubchem CID: 135618521). As per Stuart-Fox *et al*. [3], we quantified carotenoids and pteridines that have been previously reported in reptiles; carotenoids: astaxanthin, lutein/zeaxanthin, 3-dehydrolutein, canthaxanthin and beta-carotene; and pteridines: xanthopterin, sepiapterin, drosopterin, isoxanthopterin, pterine-6-carboxylic acid, pterin, 6-biopterin, neopterin and 7,8-dihydrobiopterin. As the latter six pteridines are colourless and astaxanthin, 3-dehydrolutein and canthaxanthin were absent in our samples, we report concentrations of the coloured pteridines (xanthopterin, sepiapterin and drosopterin) and the two carotenoids with detectable concentrations (lutein/zeaxanthin and beta-carotene). We extracted carotenoids, pteridines and riboflavin using a sequential liquid-liquid extraction. Riboflavin is partially extracted into the initial ‘carotenoid phase’ but can readily be recovered owing to its insolubility in acetone [25]. It was subsequently combined and quantitated with the ‘pteridine phase.’

In brief, weighed skin samples were homogenized in methanol : ethylacetate (6 : 4 v/v; containing 0.01% butylated hydroxytoluene and internal standard: trans-*β*-apo-8'-carotenal, Cayman Chemicals, USA) using a TissueLyser II system (with two 3 mm tungsten-carbide beads; Qiagen, Germany). The resulting carotenoid extract was collected following centrifugation. Pteridines were then extracted from the tissue pellet using 2% ammonium hydroxide (containing internal standard: biopterin-d3; Santa Cruz Biotechnology, USA). The carotenoid fraction was dried in a rotational vacuum concentrator (Speedvac) and washed three times with 100 μl 100% acetone (ice-cold). Dissolved carotenoids were removed, whereas acetone-insoluble riboflavin was dissolved in 2% ammonium hydroxide and combined with the pteridine fraction. The resultant ‘purified’ carotenoid fraction, and the pteridine-riboflavin fraction were dried again under vacuum and stored at −80°C until further analysis. Volumes and extraction conditions are as reported in [3] unless noted.

Carotenoids (lutein/zeaxanthin, beta-carotene) and coloured pteridines (xanthopterin, sepiapterin and drosopterin) plus riboflavin were quantified in two separate analyses on an Agilent 6490 triple quadrupole MS system with a Jet Stream electrospray ionization source coupled to an Agilent 1290 series LC system (Agilent Technologies, USA). The 6490 MS system was operating in dynamic multiple reaction monitoring, including a primary and a qualifier ion (all transitions as reported in [26]). Riboflavin transitions used were 377.1 m/z to 243 and 172.2, with collision energy set to 24 and 40 (primary and qualifier transitions, respectively). For carotenoids, the 6490 MS system was operated in positive mode with the following conditions: gas 250°C, gas flow 12 l min^–1^, nebulizer 20 psi, sheath gas 400°C, sheath gas flow 12 l min^–1^, capillary 3500 V, nozzle 0 V and high and low radio frequency (RF) at 150 and 60 V, respectively. For pteridines, the 6490 MS system was operated in positive mode: gas 250°C, gas flow 12 l min^–1^, nebulizer 50 psi, sheath gas 400°C, sheath gas flow 12 l min^–1^, capillary 1500 V, nozzle 500 V, and high and low RF at 100 and 40 V, respectively.

Chromatographic separation of carotenoids on a Syncronis C18 column (2.1 × 100 mm, 1.7 μm; Thermo Fisher Scientific, USA) was adapted from [27]. Mobile phases were A: 10 mM ammonium formate in acetonitrile : water 9 : 1 (v/v), and B: 30% isopropanol in acetonitrile. Mobile phase mix: 5 minutes 5% B, 11 minutes to 30% B, 6 minutes to 100% B and hold for 8 minutes. Flow rate: 0.4 ml min^–1^. Column temperature: 40°C. Lutein and zeaxanthin are position isomers that are not baseline separated by this method; thus, concentrations of the pair lutein/zeaxanthin were estimated from a lutein calibration curve. Retention times (RTs): lutein/zeaxanthin 6.4 minutes, beta-carotene 25.8 minutes. Chromatographic separation of pteridines and riboflavin on a Luna NH2 column (2 × 150 mm, 3 μm; Phenomenex, USA) was adapted from [28]. Mobile phases were A: 0.1% formic acid in acetonitrile, and B: 0.1% formic acid and 10 mM ammonium formate in water. Mobile phase mix: 4.8 minutes 85% B, 5.2 minutes to 50% B. RTs: drosopterin 1.6 minutes, sepiapterin 2.8 minutes, xanthopterin 5.1 minutes. Flow rate: 0.4 ml min^–1^. Column temperature: 45°C. Solvents and chemicals were obtained from Sigma-Aldrich (Merck, USA) and Thermo Fisher Scientific (USA).

Data were analysed using Agilent MassHunter Workstation Software (version B.07.00). All peak assignments were matched against commercial or purified (drosopterin) standards, confirmed with a qualifier ion and quantified against the linear range from six-point calibration curves (all *R*^2^ > 0.95). Samples and calibration standards were dissolved in acetonitrile : acetone (2 : 1 v/v) or dilute ammonium hydroxide (approx. 0.02%), respectively. Chemical standards were obtained from Sapphire Biosciences (Redfern, Australia) apart from beta-carotene (Sigma-Aldrich). Drosopterin was obtained from red eyes of *Drosophila melanogaster* (wild-type Oregon) following a two-step approach to first enrich fly heads [29] and then to extract and purify drosopterin chromatographically using Sephadex and cellulose as described by Yim *et al*. [30]. Final concentrations were normalized against tissue weight, that is, ‘pigment per gram of tissue’ referred to as ‘concentration’ throughout for brevity (table 1).

### 2.3 Statistical analysis

For the analyses of lizard samples, we compared concentrations of pigments in body regions with and without yellow using general linear mixed models (GLMM) with individual ID as a random factor to account for non-independence of samples from the same individual. Pigment concentration was the response variable; raw pigment concentrations in pmol mg^–1^ of body tissue were log-transformed to meet model assumptions. We ran a separate model for each of the main yellow or red pigments detected: riboflavin, xanthopterin, drosopterin and lutein. Data were analysed in R v4.4.0 [31] using the lme4 package [32]. We only performed statistical comparisons for the lizards, for which we sampled from multiple body regions from two individuals. We did not do statistical tests for the corn snakes, because although we had replicate samples, these were from a single individual per morph.

### 2.4 Transmission electron microscopy

Corn snake samples were processed as described by Ullate-Agote *et al*. [33]. Micrographs of 50 nm transversal ultrathin sections were taken with a transmission electron microscope (Philips CM100; Thermo Fisher Scientific).

## 3 Results

Concentrations of riboflavin were significantly higher in the yellow dorsal region compared with the lateral region of the Indian rock agama (*χ*_1_^2^ = 18.31, *p* < 0.0001; figure 1A; table 2). Carotenoids (yellow lutein and orange beta carotene) were present in very small concentrations (table 1) and did not differ significantly between body regions (table 2). Comparison with carotenoid levels in the cream patches of other species in our dataset (table 1) suggests that carotenoid pigments at these concentrations may contribute little to skin coloration and instead represent background cellular levels. Xanthopterin, a yellow pteridine, was present in relatively high amounts (table 1), but these were not significantly different between dorsal and lateral colour patches.

In the pink-lined dragon, concentrations of riboflavin were higher in the light yellow-brown dorsal region and yellow lateral stripe than the white ventral region (*χ*_1_^2^= 11.58, *p* = 0.0007; figure 1B; tables 1 and 2). Unsurprisingly, xanthopterin and drosopterin concentrations also differed significantly between these body regions (tables 1 and 2) because the white ventral region largely lacks coloured pigments. These results suggest that riboflavin, together with pteridine pigments, contributes to the yellow-brown dorsal coloration in this species.

The skin of wild-type corn snakes contained little carotenoids but high concentrations of pteridines (figure 1C; table 1). The concentration of pteridines (xanthopterin and drosopterin) was substantially higher in the dark brick-red blotches and lighter brick-red background than the orange (i.e. yellower) lateral region, whereas riboflavin concentrations were very high in the lateral samples and modest in the blotch and background samples. Riboflavin concentrations were higher in the lateral samples of wildtype corn snakes than any other samples from any of the species we measured.

In wild-type skin, xanthosomes contain pterinosomes with characteristic concentric lamellae associated with pteridine pigments (figure 1F; [33]). By contrast, xanthosomes in the Snow morph are enlarged, contain amorphous material and lack these lamellae (figure 1G). The impacted xanthosomes in the Snow morph correspond to extremely low concentrations of pteridine pigments (yellow xanthopterin and red drosopterin), compared with the wild-type (figure 1D; table 1). As for the wild-type, carotenoids do not contribute to coloration in Snow corn snakes, being present in trace or background concentrations (table 1). We detected high concentrations of riboflavin in the yellow blotches and lower concentrations of riboflavin in the cream background (figure 1D; table 1), indicating that riboflavin is sufficient to produce yellow skin coloration in this species.

Overall, riboflavin concentrations were strongly associated with yellower hues across both lizard species and the Snow morph of the corn snake, as confirmed by spectrometry (figure 2).

## 4 Discussion

In this study, we provide quantitative, within-individual comparisons of pigment concentrations in colour patches that differ in yellow hues in several reptile species. This includes the Snow morph of the corn snake, in which we can isolate the contribution of riboflavin in the absence of contributions of pteridine and carotenoid pigments to skin coloration. Our results show that riboflavin is an active component of yellow coloration in the species we examined and is sufficient to produce yellow skin hues in the Snow morph of the corn snake. Together, our results highlight the need to consider riboflavin, along with pteridines and carotenoids, in studies of pigmentation in reptiles and potentially other ectothermic vertebrates. Indeed, in some reptile species, riboflavin may be a stronger contributor to skin coloration than carotenoids. Like carotenoids, riboflavin cannot be synthesized de novo by vertebrates and must be acquired through the diet, raising the possibility that its availability may be limiting. Moreover, riboflavin is a potent antioxidant and an essential cofactor in cellular metabolism [34]. These parallels suggest that riboflavin-based coloration could be subject to resource allocation trade-offs and condition dependence [35]. If so, riboflavin pigmentation may function as an honest signal of individual quality.

In snakes, riboflavin concentrations in yellow skin appear to be particularly high [15–17], and our quantitative data for the corn snake indicate that it constitutes the dominant pigment in yellow patches. In the two lizard species we examined, riboflavin concentrations were higher in skin with yellow hues, but xanthopterin, a yellow pteridine, was nonetheless the dominant yellow pigment in terms of absolute concentration. Differences between snakes and lizards in the relative importance of riboflavin as a skin coloration could be related to diet. Riboflavin may be more readily available in the diet of snakes than lizards because there are relatively high amounts of riboflavin sequestered in animal tissue, such as liver, muscle and eggs [36]. By contrast, yellow carotenoids, which are sequestered from plants [5], may be less readily available in the diet of snakes than other groups such as lizards, fish or turtles. We hypothesize that riboflavin and pteridines, rather than carotenoids and pteridines, may be the dominant yellow to red pigments across snakes.

In ectothermic vertebrates (fishes, amphibians and reptiles), yellow to red pteridine and/or carotenoid pigments are found within xanthophore pigment cells. Xanthophores are often found in the upper-most layer of a complex layered system of pigment cells, the dermal chromatophore unit, responsible for skin coloration [37]. The layers comprise different chromatophore types, which are conserved across ectothermic vertebrates, though the presence, density and arrangement of these pigment cells vary among colour patches [37]. This layered system combines light absorption by pigments and light scattering/reflection by integumental nanostructures. Light that is not absorbed by pigments in xanthophores interacts with other chromatophores, namely, iridophores containing reflective guanine platelets and melanophores containing densely packed melanin vesicles. Thus, the overall colour or skin reflectance arises from multiple pigments and a complex interaction between pigment cells. Although carotenoids and pteridines have been localized to specialized organelles, the xanthosomes, within xanthophore pigment cells, the location of riboflavin is uncertain.

Our electron micrographs showed that Snow xanthosomes are heavily impacted and associated with very low amounts of pteridine pigments, yet riboflavin levels are high. It is not clear whether riboflavin is localized to specific pigment cells or found in the extracellular matrix. Indeed, electron microscopy cannot shed light on the localization of riboflavin, just as the presence of specific pteridines cannot be inferred solely from micrographs. At present, the sensitivity and spatial resolution of *in situ* pigment analyses (e.g. time-of-flight secondary ion mass spectrometry (ToF-SIMS)) are insufficient to localize riboflavin to specific cells or organelles. Nevertheless, studies in cultured cells show that riboflavin can localize to intracellular acidic vesicular compartments following uptake [38]. Our data suggest that riboflavin does not depend on properly formed xanthosomes. It may instead be present in other intracellular compartments across diverse skin cell types, not limited to chromatophores, which remain to be identified.

Similarly, the genetic basis of colour variation linked to riboflavin concentrations in ectothermic vertebrates remains unclear. In ball pythons (*Python regius*), the absence of yellow pigmentation (axanthism) is linked to the gene coding for guanosine triphosphate (GTP) cyclohydrolase II (*gch2*), which catalyses the first rate-limiting step in riboflavin biosynthesis in bacteria [39]. However, its role in animals, which cannot synthesize riboflavin, is unclear. The enzyme GTP cyclohydrolase II shares affinity with GTP cyclohydrolase I, which is involved in the biosynthesis of folic acid and biopterin, and genes for both enzymes are markers for xanthophore development in fishes [40,41]. The specific pigments that underlie yellow coloration in ball pythons have not yet been identified. Conversely, in the wall lizard (*Podarcis muralis*), riboflavin concentrations appear to be higher in colour morphs with orange coloration than those without [9]. However, genetic differences between morphs were confined to changes in regulatory regions near genes associated with pterin (sepiapterin reductase) and carotenoid (beta-carotene oxygenase 2) metabolism [9]. Thus, an outstanding question is the molecular mechanisms of transport and deposition of riboflavin in specific skin colour patches.

Our findings raise important questions about the generality of riboflavin’s contribution to skin col-oration, and the mechanisms and consequences. Does riboflavin contribute to skin coloration primarily in species where carotenoids play little role? How important is the contribution of riboflavin relative to pteridines? Where and how is riboflavin sequestered in skin tissue? How is riboflavin linked to diet and what are eco-evolutionary implications? By showing that riboflavin may be an important pigment contributing to yellow coloration in some snakes and lizards, we hope to encourage the inclusion of riboflavin in quantitative pigment analyses across a broader phylogenetic range of ectothermic vertebrates.

## Figures and Tables

**Table 1:** Yellow to red pigment concentrations in skin of the Indian rock agama, the pink-lined dragon and two colour morphs, wild-type and Snow, of the corn snake. (Note: values are mean and s.e. (± s.e.) of absolute concentrations in pmol mg^–1^ of skin tissue. The amounts of beta-carotene and sepiapterin are negligible in all samples. Note the high concentration of riboflavin in the Snow morph of the corn snake.)

speciesand body region	patch colour	riboflavin yellow flavin	xanthopterin yellow pteridine	sepiapterin yellow pteridine	drosopterin red pteridine	lutein yellow carotenoid	beta carotene orange carotenoid
Indian rock agama dorsal	yellow to red	48.533 ± 9.540	343.533 ± 154.602	0	12 ±5.537	3.123 ± 1.678	0.017 ± 0.012
Indian rock agama lateral	red to black	21.15 ± 11.65	456.95 ± 320.15	0.7 ± 0.7	45.6 ± 39.5	1.6 ± 1.59	0.01 ± 0.01
pink-lined dragon dorsal	yellow-brown	121.675 ± 50.334	562.212 ± 141.591	0.28 ± 0.162	322.76 ± 178.276	7.445 ± 1.486	0.378 ± 0.036
pink-lined dragon ventral	cream	14.505 ± 4.175	46.9 ± 24.22	0	7.485 ± 3.005	3.551 ± 0.124	0.262 ± 0.032
corn snake—wild-type blotch	brick red	47.41 ± 15.76	301.74 ± 38	0.91 ± 0.78	296.815 ± 207.325	0	0.009 ± 0.004
corn snake—wild-type background	dark brick red	100.055 ± 22.065	839.98 ± 682.57	0.885 ± 0.575	436.27 ± 343.22	0	0.007 ± 0.001
corn snake—wild-type lateral	orange	1212.89 ± 605.35	27.28 ± 0.82	0	14.95 ± 4.98	0	0.0065 ± 0.0005
corn snake—Snow blotch	yellow	454.05 ± 278.45	10.65 ± 9.25	0	0	1.06	5.38
corn snake—Snow background	cream	149.2 ± 51.8	7.75 ± 4.75	0	0	3.125 ± 1.365	0.145 ± 0.145

**Table 2:** GLMMs testing for differences in riboflavin and pigment concentrations between body regions with and without yellow for agamid lizard species. (Note: we ran a separate model for each pigment in the table. *** denotes *p* < 0.001.)

species	molecule	*χ* ^2^	d.f.	*P*
Indian rock agama	riboflavin	18.309	1	1.878e–05***
	xanthopterin	0.1194	1	0.7297
	drosopterin	0.0376	1	0.8462
	lutein	0	1	0.995
pink-lined dragon	riboflavin	11.576	1	0.000668***
	xanthopterin	19.877	1	8.258e–06***
	drosopterin	11.135	1	0.0008472***
	lutein	3.0765	1	0.07943

## Data Availability

The data associated with this study is available on Figshare [42].
